# Opportunistic Infections among newly diagnosed HIV patients in the largest tertiary facility in Ghana

**DOI:** 10.5334/aogh.4149

**Published:** 2024-02-13

**Authors:** Peter Puplampu, Olive Asafu-Adjaye, Marian Harrison, John Tetteh, Vincent Jessey Ganu

**Affiliations:** 1 Department of Medicine and Therapeutics University of Ghana Medical School, Accra, Ghana; 2 Ghana Health Service, Ghana; 3 Department of Internal Medicine, Ghana Police Hospital, Ghana; 4 Community Health Department, University of Ghana Medical School, Ghana; 5 Department of Medicine and Therapeutics, Korle-Bu Teaching Hospital, Ghana

**Keywords:** Opportunistic infections, WHO HIV clinical stage III & IV

## Abstract

**Background:**

Opportunistic infections (OIs) among newly diagnosed HIV patients are a marker for inadequateness of HIV awareness and testing. Despite global efforts at creating awareness for early detection, late HIV diagnosis and its associated OIs still exist. This study sought to determine the prevalence and patterns of OIs and associated factors among newly diagnosed HIV patients in Ghana.

**Methods:**

A retrospective study using data extraction was conducted among 423 newly diagnosed HIV patients aged ≥18 years at the Korle-Bu Teaching Hospital from July 1^st^ 2018 to December 2019. Multivariate logistic regression was adopted to assess factors associated to OIs. Analysis was performed using SPSS version 16, and p-value < 0.05 was deemed significant.

**Results:**

The mean age of patients with a new HIV diagnosis was 40.15 ± 11.47 years. Male versus female sex differential was 30.3% and 69.7%, respectively. The prevalence of OIs among newly diagnosed HIV patients was 33.1% (95% CI = 34.6–44.1). About 70% (120/166) of patients with OIs were classified into WHO clinical stage III and IV. The most common OIs were candidiasis (oro-pharyhngeal-esophageal) (36.9%), and cerebral toxoplasmosis (19.9%). The odds of an OI at the time of HIV diagnosis among females was 51% lower than in males (aOR = 0.49, 95% CI = 0.28–0.86). Being employed increased the odds of OIs by 2.5 compared to the unemployed (aOR = 2.5; 95% CI = 1.11–5.61). Participants classified as World Health Organization (WHO) HIV clinical stage III and IV were 15.88 (95% CI = 9.41–26.79) times more likely to experience OIs.

**Conclusion:**

One in three patients newly diagnosed with HIV presented with an opportunistic infection, with men more likely to experience such infections. Significant attention should be given to improving case-finding strategies, especially among men.

## Introduction

Opportunistic infections (OIs) have accounted for the majority of illnesses and death among HIV-infected individuals since the HIV epidemic began in the 1980s, with Sub-Saharan Africa and Asia bearing the brunt of the burden [1,2]. However, this has changed over the years with the development of new HIV testing methods and Highly Active Antiretroviral Treatment (HAART), which have significantly reduced AIDS-related morbidity and death worldwide, including those from opportunistic infections [3]. The global introduction of the “treat all policy” in 2016 was another effort to ensure infected persons were put on treatment immediately [4]. Tracking these developments has also been made easier by global targets set by UNAIDS, which have transitioned from 90–90–90 (90% of all people living with HIV will know their HIV status, 90% of all people with diagnosed HIV infection will receive sustained antiretroviral therapy, and 90% of all people receiving antiretroviral therapy will have viral suppression by 2025) to 95–95–95 (95% of all people living with HIV will know their HIV status, 95% of all people with diagnosed HIV infection will receive sustained antiretroviral therapy, and 95% of all people receiving antiretroviral therapy will have viral suppression by 2030) [4].

Over the years, Ghana has adopted the treat all policy and has implemented several strategies to improve testing in a bid to improve AIDS-related morbidity and mortality, especially those from opportunistic infections [3,5,6,7]. Some of these strategies embarked by the country under the supervision of National AIDS Control Program and the Ghana AIDS Commission to improve testing by reducing AIDS-related mortality and morbidity include Know Your Status’ (KYS) campaign, Know Your Rights, and Know Your Response, in addition the various standard operating guidelines that have been applied to reduce prevalence of HIV within the country [3,5,6,7,8,9,10,11]. Despite all these measures, the Sub-Saharan region and countries burdened with HIV/AIDS still suggest that AIDS-related opportunistic infections (including tuberculosis) remain high [1,12,13,14]. Many of these studies varied with regard to prevalence rates, distribution of the type of infections, and epidemiological characteristics of persons with opportunistic infections, and mostly focused on patients on HAART, with a few on newly infected patients [1,13,14,15,16,17].

A Nigerian study focusing on newly diagnosed HIV patients, the overall prevalence of opportunistic infections in newly diagnosed patients aged was 46.6%, with oral candidiasis being the most common OI. Significant factors associated with opportunistic infections were being 50 years or older, having a CD4 count lower than 350, and having a hepatitis C virus co-infection [17]. Within Ghana, there are very limited studies with regard to prevalence and distribution of opportunistic infections in newly infected persons and in the era of the “treat all policy.” This study sought to describe the prevalence, socio-demographic, and epidemiological/clinical characteristics of opportunistic infections among newly diagnosed HIV adult patients at the Korle-Bu Teaching Hospital in southern Ghana.

## Methods

### Study design and site

A retrospective study design with a data extraction approach was conducted over an 18-month period from July 1^st^ 2018 to December 2019 at the Fever’s Unit (Infectious disease unit) of the Korle Bu Teaching Hospital (KBTH). The hospital serves as the main national referral center for the care of persons living with HIV in southern region of Ghana.

### Data collection and tools

Patients who accessed services at the Fevers Unit for the period under review and were newly diagnosed of HIV were identified in the electronic database of patients’ records. Unique study serial numbers issued to each new HIV-diagnosed patient at first presentation were collected from the database and used to identify the medical records of these patients. Participants’ names were not included in any part of the study to ensure patient confidentiality and privacy.

Data was extracted from medical records using a structured questionnaire. Data extracted included socio-demographic parameters such as age, sex, married status, educational status, and occupation. Clinical data extracted included WHO clinical stage, HIV type, body mass index (BMI), duration of HIV diagnosis, and OI symptom duration. CD4 was not included in the data collected, as it was unavailable due to CD4 testing no longer being part of the routine investigations for PLHIV as per the national guidelines.

### Operational definitions

**Opportunistic infection** – defined as infections caused by various microorganisms that cause disease in people with compromised immune systems (as in HIV), many of which would not ordinarily cause disease in people with competent immune systems [18].

### Data analysis

Data was stored in Microsoft Excel files and later exported to SPSS version 16 (Atlanta, USA) for analysis. Statistical analysis was primarily descriptive with categorical data presented as frequencies and percentages. Univariate and multivariate logistic regression analysis were carried out to assess factors associated with OIs. P-value < 0.05 was considered significant.

### Ethical approval

Study approval was obtained from the Institutional Review Board (IRB) of KBTH with approval identification number KBTH-IRB/00093/2020.

## Results

### Characteristics of study participants and prevalence and distribution pattern of opportunistic infections

A total of 423 newly diagnosed Persons Living with HIV (PLHIV) were identified from the electronic database during the time period and all were included in the study with 69.7% (295/423) being female. The average age of our participants was 40.15 ± 11.47 years, with an age range of 18 to 60+ years (Table 1). In terms of marital status, 41% of participants (176) were single, while 39.9% were married. Thirty-seven percent of the study participants had received an education up to the tertiary level. The overall prevalence of opportunistic infections was 39.2% (95% CI = 34.6–44.1), with around 90% having at least one opportunistic infection.

**Table 1:** Presence of opportunistic infection by sociodemographic characteristics among newly diagnosed HIV/AIDS patients at the Fevers Unit, Korle Bu Teaching Hospital, 2018–2019 (n = 423).


SOCIODEMOGRAPHIC CHARACTERISTICS	TOTAL n(%)	CATEGORY	OPPORTUNISTIC INFECTIONS (%)		P-VALUE

			YES	NO	

**Sex**	128	Male	55(43.0)	73(57.0)	0.326

	295	Female	111(37.9)	182(62.1)	

**Age (years)**	5	<20	1(20.0)	4(80.0)	0.337

	77	20–29	23(30.3)	53(69.7)	

	137	30–39	57(41.9)	79(58.1)	

	115	40–49	50(43.5)	65(56.5)	

	64	50–59	23(35.9)	41(64.1)	

	25	60+	12(48.0)	13(52.0)	

**Marital Status**	15	Cohabited	6(40.0)	9(60.0)	0.979

	31	Divorced	13(41.9)	18(58.1)	

	169	Married	62(36.9)	106(63.1)	

	176	Never married	72(41.1)	103(58.9)	

	2	Separated	1(50.0)	1(50.0)	

	30	Widowed	12(40.0)	18(60.0)	

**Level of education**	46	No formal education	18(39.1)	28(60.9)	0.140

	42	Primary	21(50.0)	21(50.0)	

	108	Secondary	44(40.7)	64(59.3)	

	157	Tertiary	63(40.6)	92(59.4)	

	68	Vocational	18(26.5)	50(73.5)	

**Occupation**	70	Unemployed	46(65.71)	24(34.3)	0.453

	29	Student	18(62.1)	11(37.9)	

	195	Self-Employed	108(55.4)	87(44.6)	

	118	Employed	71(60.2)	47(39.8)	

	9	Retired	4(44.4)	5(56.6)	

	2	Other	0(0.0)	2(100.0)	

**Entry Point**	82	External referral	43(52.4)	39(47.6)	0.001

	278	Internal referral	110(39.9)	166(60.1)	

	63	Walk ins	13(20.6)	50(79.4)	

**Number of OIs**	256	0	0(0.0)	256(100.0)	0.000

	140	1	140(100.0)	0(0.0)	

	24	2	24(100.0)	0(0.0)	

	3	3	3(100.0)	0(0.0)	

**WHO stage**	193	Stage I	15(7.8)	176(92.2)	0.000

	59	Stage II	31(52.5)	28(47.5)	

	96	Stage III	59(61.5)	37(38.5)	

	73	Stage IV	61(83.6)	12(16.4)	


### Incidence and types of opportunistic infections of newly diagnosed HIV/AIDS patients

The most common opportunistic infection was candidiasis (oro-pharygeal-esophageal), accounting for 36.9% of the opportunistic infections present, followed by cerebral toxoplasmosis (19%) (Table 2).

**Table 2:** Incidence and Types of Opportunistic Infections of Newly Diagnosed HIV/aids patients At The Fevers unit, Korle Bu Teaching Hospital, 2018–2019.


TYPE OF OI	FREQUENCY (N)	PERCENTAGE

**Anogenital Warts**	1	0.6%

**Candidiasis (oro-pharyngeal-esophageal)**	63	36.9%

**Carbuncle**	1	0.6%

**Cerebral Toxoplasmosis**	33	19.9%

**Chronic Gastroenteritis**	9	5.4%

**Cryptococcal Meningitis**	1	0.6%

**Furunculosis**	1	0.6%

**Genital Herpes**	4	2.4%

**Herpes Zoster**	1	0.6%

**Lymphoma**	2	1.2%

**Oral hairy leukoplakia**	1	0.6%

**Pneumocystis Jiroveci Pneumonia**	6	3.6%

**Pneumonia**	8	4.8%

**Pruritic Papular Dermatitis**	18	10.8%

**Pulmonary Tuberculosis**	11	6.6%

**Syphilis**	3	1.8%

**Tuberculous meningitis**	1	0.6%

**Vulvovaginitis**	2	1.2%

**Total**	166	100.0%


### Association between socio-demographic and clinical characteristics and opportunistic infections

The results of multiple logistic regression models fit to determine the independent factors associated with opportunistic infections (OIs) in newly diagnosed patients are presented. Demographic variables (marital status, BMI, entry points, and education) were not associated with OIs. Significant risk factors for the presence of an opportunistic infection at the time of HIV diagnosis included the male gender, occupation, and WHO stage III and IV. The odds of OIs reduced by 51% among females compared with males (aOR = 0.49; 95% CI = 0.28–0.86). Being a student, self-employed, and employed, significantly increased the odds of OIs compared with unemployed participants [aOR(95% CI) = 3.78(1.17–12.21), 3.63(1.47–12.21) and 2.50(1.11–5.61) respectively]. Participants that presented with an illness classified as WHO stage III and IV were over 16 times more likely to experience OIs compared with those classified as WHO stage I–II (aOR = 15.88, CI = 9.41–26–79) (Table 3).

**Table 3:** Predictors of presence of opportunistic infections among newly infected HIV/AIDS patients, Korle Bu Teaching Hospital 2018–2019.


SOCIODEMOGRAPHIC AND CLINICAL CHARACTERISTICS	CATEGORY	cOR(95% CI)	aOR(95% CI)	P-VALUE (aOR)

**Sex**	Male	1	1	

	Female	0.74[0.49–1.14]	0.49[0.28–0.86]	0.013

**Occupation**	Unemployed	1	1	

	Student	1.17[0.48–2.87]	3.78[1.17–12.21]	0.026

	Self-Employed	1.54[0.87–2.87]	3.63[1.71–12.21]	0.001

	Employed	1.27[0.68–2.35]	2.50[1.11–5.61]	0.026

	Retired	2.39[0.58–9.75]	3.26[0.55–19.18]	0.192

	Other			

**Marital status**	Cohabited	1	1	

	Divorced	0.1.60[0.45–5.58]	1.64[0.34–7.86]	0.536

	Married	0.97[0.33–2.86]	1.41[0.37–5.40]	0.612

	Never married	1.04[0.35–3.07]	1.68[0.44–6.45]	0.446

	Separated	1.50[0.08–28.89]	0.92[0.03–26.49.]	0.961

	Widowed	1.00[0.28–3.54]	1.61[0.33–1.79]	0.553

**WHO stage**	Stage I–II	1	1	

	Stage III–IV	12.45[7.80–19.88]	15.88[9.41–26–79]	<0.001


## Discussion

Opportunistic infections are one of the main causes of morbidity and mortality in immunocompromised individuals due to impaired immune systems, particularly in persons living with HIV/AIDS. This study determined the prevalence, sociodemographic, and epidemiological characteristics for OIs in newly diagnosed PLHIV in the largest tertiary hospital in Ghana. Approximately 70% of study participants were women, which is consistent with data from the 2020 national and sub-national estimates [5]. These results were also similar to studies conducted in Cameroon and Guinea Bissau [13,14,15,16,17], and emphasizes the point that women are more likely to know their status and more likely to seek treatment thus making up the bulk of the study populace. Furthermore, this study found that being female signifies lesser odds of developing opportunistic infections. This serves as a stark reminder that more needs to be done to reach males of all ages, mandating more targeted, strategic programs that focus on men getting to know their status [19].

Patients with OIs were mostly within age groups of 30–39 and 40–49 years. This contrary to a study done in Nigeria that found newly diagnosed patients with OIs to be aged 50 and above [17]. Younger adults may engage in risky sexual behavior, leading to undetected infections until symptoms appear. In addition to this, they may suffer from internal and external stigma, resulting in poor adherence.

In spite of various investments toward increasing HIV testing opportunities available within the Sub-Saharan region and the country, a majority of newly diagnosed individuals became aware of their status only after they developed opportunistic infections in the late stages [20]. This is evident, as the overall prevalence of OIs was 33.1%. Though prevalence is lower than what was documented in Nigeria (46.6%), this figure is still quite high as OIs tend to present when PLHIV are in advanced stages [2,3,5,17]. Candidiasis (oro-pharyngeal-esophageal) was the most common OI, closely followed by cerebral toxoplasmosis. This is similar to the results of the study in Togo and Nigeria [17,21], but slightly differs from the global picture where CNS opportunistic infections are the most common [22]. In our setting, limited capacity in diagnostics or screening for CNS OIs may account for the reduction in the number of CNS OI cases reported. Despite this, CNS OIs were the second most common OIs amongst our study population.

Our study revealed a 3.8 fold increase in the odds of presenting with OI among students, a 3.6 fold increase among the self-employed, and a 2.5 fold increase among the employed, compared with the unemployed. Possible reasons for this include the stress levels involved with schooling and employment, which tends to activate macrophages resulting in their subsequent depletion [23]. Frequent engagement with diverse people at school and work predisposes them to infections or diseases that deplete their macrophages and makes them susceptible to opportunistic infections compared to those unemployed [23]. Effective health education and promotion activities should be conducted to get people to test and know their HIV status for early initiation of treatment to avoid OIs and their complications.

In this study, the highest significant risk factor for developing opportunistic infections was WHO clinical stage III and IV. Those in stages III and IV were found to be 13 times more likely to develop infections compared to those in stages I and II (OR10.63, p = 0.000). In a comparable study, patients in WHO stages III and IV were also found to be 10 times more likely to develop infections compared to those in stages I and II (OR = 9.15, p < 0.0001) [24]. In this cited study, however, the patients seen had already been initiated on HAART. Thus, though significant, a lesser number of the study population were classified as WHO III&IV.

The most likely explanation to this may be that patients in stages III or IV are also likely to have high viral load and low CD4+ count, both of which are risk factors for development of opportunistic infections as in a study in Nigeria, which focused on CD4+ count [17]. Participants with OIs were also mostly single, and were referred from peripheral facilities to the Fevers unit. All these serves as a stark reminder that case finding strategies may need to be intensified, especially within the communities to ensure the people know their HIV status as this sets the stage for timely intervention.

## Limitations of the Study

Due to the possibility that some patients’ clinical information and test results from the laboratory were lost, this study, like other retrospective studies, is prone to information bias. Further research may want to concentrate trends in OI in patients already on HAART.

## Conclusion

The prevalence of opportunistic infections was high among newly diagnosed PLHIV. More attention should be paid by national HIV program mainstreaming managers and policymakers to the male sex and individuals with advanced cases of HIV to re-examine and intensify existing case identification methodologies, comprehensive HIV care and support activities.
